# Double-Tension Wire Management of Nonunion Patella with Severe Quadriceps Contracture

**DOI:** 10.1155/2018/1364129

**Published:** 2018-09-06

**Authors:** Rohan Bhimani, Preeti Singh, Fardeen Bhimani

**Affiliations:** ^1^Department of Orthopaedics, 11^th^ Road, Khar (West), Hinduja Healthcare Surgical, Mumbai 400052, India; ^2^Department of Orthopaedics, Osmania General Hospital, Hyderabad 500012, India; ^3^Department of Orthopaedics, Bharati Hospital, Pune 411043, India

## Abstract

**Introduction:**

Nonunion patella with quadriceps contracture is an unusual orthopaedic finding. Very few cases have been recorded in the past with this complication. We present a case of a 40-year-old male with nonunion patella with quadriceps contracture secondary to trauma*. Case Report.* A 40-year-old male with posttraumatic nonunion patella with quadriceps contracture since 6 months presented with complaints of defect in the left knee with restriction of movements. X-ray of the left knee confirmed our findings. He underwent quadricepsplasty with double-tension band wiring for the patella followed by rigorous physiotherapy to achieve the current level of the knee flexion of 110 degrees.

**Conclusion:**

We conclude that quadricepsplasty with tension band wiring and neutralization wire is one of the good modalities of treatment for a nonunion patella associated with quadriceps contracture.

## 1. Introduction

Fractures of the patella contributes to 1% of all skeletal injuries [1]. The anterior subcutaneous location of the patella makes it vulnerable to direct trauma. Transverse fracture pattern of the patella is a common form of presentation in clinical practice. Delayed presentation of displaced fracture of the patella is a common presentation in practice. Majority of the times when such cases present, the fragments are grossly displaced. Furthermore, there are soft tissue contractures like quadriceps, retinaculum, internal ligaments of the knee joint, associated knee joint stiffness, and extensor lag in these patients. The major hurdle is to bring the fracture fragments together and restore the extensor mechanism either by bone to bone or bone to tendon union. It is essential to maintain the length of the contracted tissues by allowing further flexion of the knee. There are three different school of thoughts in terms of the management for such complex fracture presentation. First school of thought is to go for conservative management with knee ROM exercises. The second group recommends single-stage procedure in which mobilization of the proximal fragment, followed by fixing with the lower fragment using V-Y or Z-plasty and achieving fractional lengthening [2]. The third group opines the use of preoperative traction to the proximal fragment using pins or Ilizarov method to approximate the fragments and then fixing the fragments. Thus, double-stage surgery is carried out [2]. In our case, we used single-stage procedure whose results were encouraging.

## 2. Case Report

**Figure 1:**
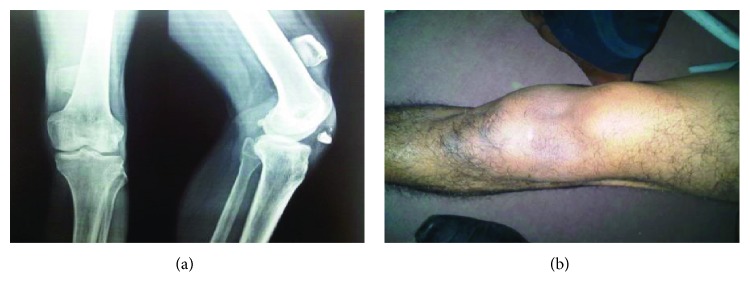
Preoperative clinical and radiological images showing proximal fragment in the distal third of the thigh (a, b).

A 40-year-old male presented with complaints of instability and defect in his left knee since 6 months. The patient gave a history of trauma 6 months back and did not take any treatment for this. Clinically, anterior defect was present over the left knee with visibility of intercondylar articulating surfaces of the tibia and femur. Swelling was seen in the anterior aspect of the left distal third thigh, which, on palpation, was the superior part of the patella. The lower pole of the patella was palpable just above the left tibial tuberosity (Figure 1). The X-ray of the left knee confirmed that the superior fragment of the patella was present in the distal third aspect of the thigh and the lower fragment close to the tibial tuberosity (Figure 1).

**Figure 2:**
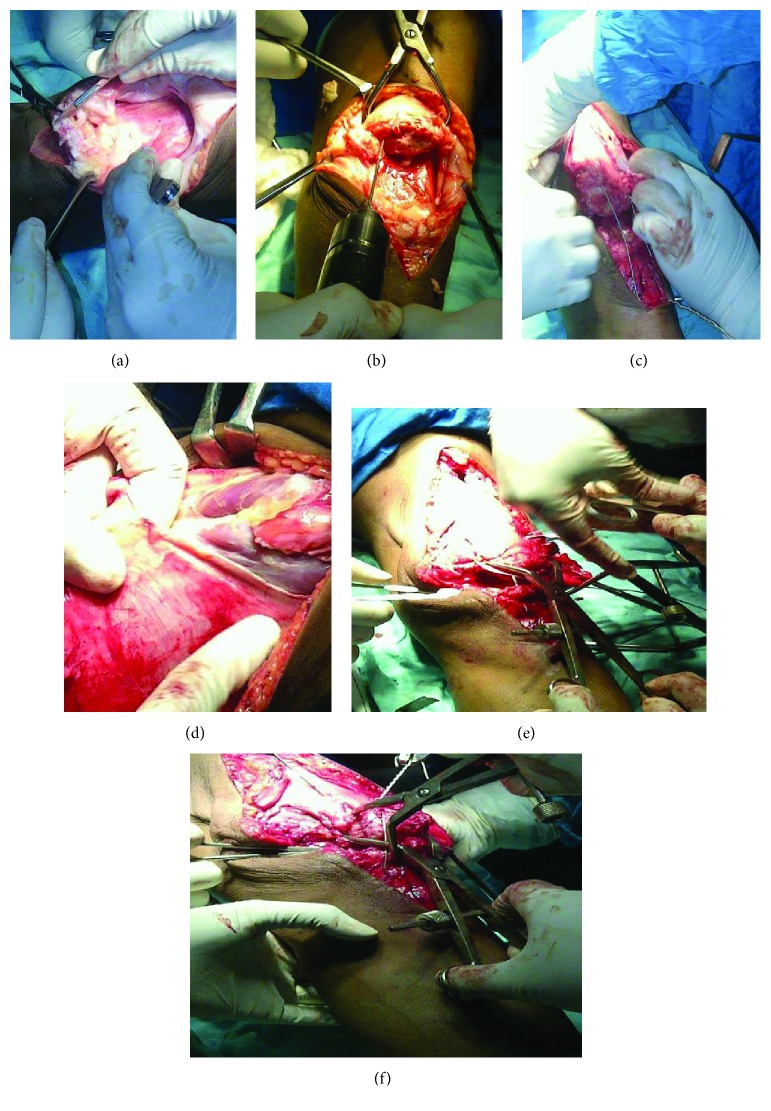
Intraoperative images of quadricepsplasty, reduction, and fixation of the patella (a–f).

The patient underwent surgery by anterior approach where quadricepsplasty and tension band wiring for the patella were performed after bringing the superior fragment down (Figure 2). Another tension band wire was passed through the neutralization hole made just posterior to the tibial tuberosity and the retinaculum was repaired.

**Figure 3:**
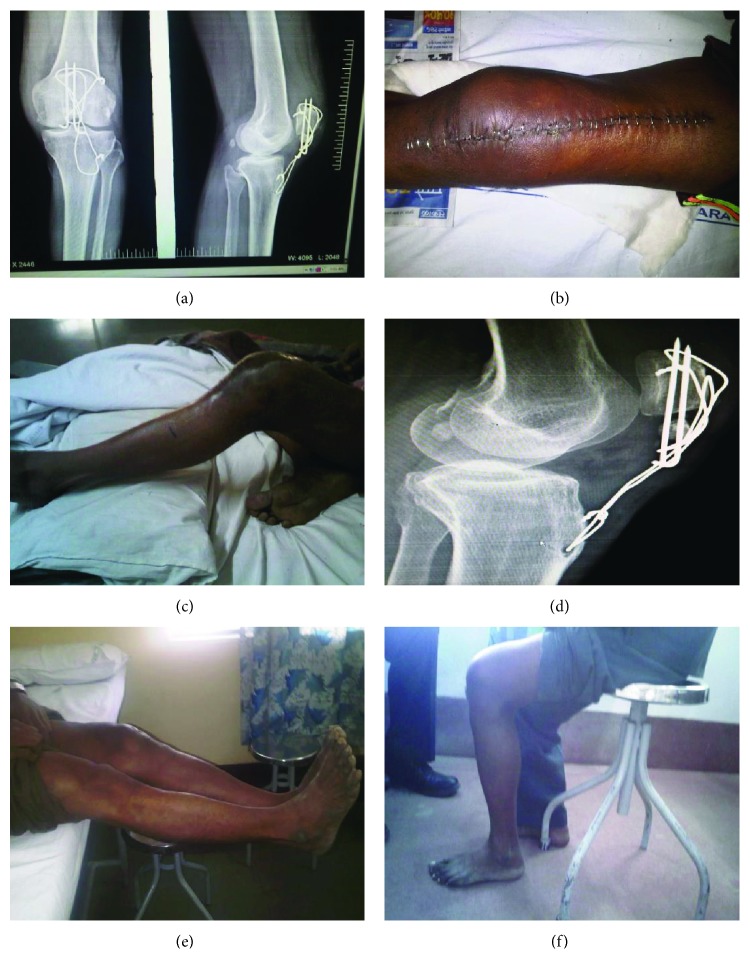
Postoperative X-ray, suture line, current X-ray, and present knee range of movement (a–f).

During the immediate postoperative period, the patient was started on dynamic quadriceps strengthening and active straight-leg-raising exercises. After suture removal, continuous passive motion for his knee was added. On discharge, the range of knee motion was from 5 degrees of extension lag to 40 degrees of flexion. At 6 weeks of postoperative follow-up, the patient had a 5- to 90-degree knee motion. The range of motion improved to 0–110 degrees at 3 months follow-up (Figure 3).

## 3. Discussion

The aim of reporting this case is to highlight the double-tension wire management and use of single-stage double-tension wire management of an old neglected nonunion patellar fracture. In the procedure, the proximal fragment was mobilized and fixed with the lower fragment using V-Y plasty and double-tension band wires. The objective during the surgery was to achieve fractional lengthening in order to prevent quadriceps lag in patients of nonunion of patellar fractures with large gaps between the fracture fragments. The rate of nonunion in patellar fractures is about 2.7% [3]. There are few cases reported in the management of such fractures. All of these cases of displaced fracture of the patella required operative intervention. The patients generally land up with a gap nonunion due to ignorance as they walk full-weight-bearing post fracture without surgical intervention. The normal tensile force across a patella is around 3000 N, which increases up to 6000 N in athletes. The generated patellofemoral compressive forces are three times greater than that of the body weight during routine daily activities and may exceed seven times the body weight while climbing stairs and squatting. These forces only act on the proximal pole in fractures associated with tears in the medial and lateral expansions. The integrity of the medial and lateral expansions along with the anterior fascia lata and Sharpey's fibres allows active extension of the knee after patellar fracture. Unopposed passage of these forces, as in our case, allows a continuous increase in the gap between the fragments leading to the contracture of the proximal quadriceps mechanism. The available supporting literature on such presentation is shown in Table 1.

The problem with double-stage surgeries is that the presence of Ilizarov or skeletal traction poses a mental trauma to the patient along with surgical complications like bone weakening, pin loosening, pin tract infection, and prolonged duration of treatment. Yet double-stage surgeries have been reported with good results as shown by Dhar and Mir [7].

We opted for single-stage procedure, i.e., proximal fragment mobilization and fixation with the lower fragment/patellar tendon using V-Y/Z-plasty and achieving fractional lengthening. The supplementary fixation with neutralization wire in the region of the patellar tendon not only acted as an internal bracing during the initial rehabilitative period but also as a compressive force that shortens an already contracted patellar tendon. In addition, it also helps in securing the reduction by decreasing the tensile forces, which are exerted by the quadriceps muscle during knee flexion. One major risk with this procedure is overtightening of the neutralization wire resulting in the patella baja. In order to ensure the correct length of the neutralization, an image intensifier should be used intraoperatively to assess and compare the distance of the tibial tuberosity and the distal pole of the patella of the contralateral knee. Moreover, biomechanical assessment of the tension band wire shows that it fails at maximum load of about 695 N after osteosynthesis of the patella. Other methods of treatment like conservative technique and patellectomy are also practiced. In the conservative method, the stability and alignment of fracture fragments are confirmed at 60 degrees of flexion under an image intensifier with no dislocation. The initial restriction of knee flexion is provided by a suitable orthosis, which is gradually increased during the course of treatment. The major risk of conservative management is loss of full extension and stiffness of the knee, which are undesirable especially in a young patient. Patellectomy is another commonly practiced salvage procedure [3, 8]. This results in devastating problems like long periods of rehabilitation, anterior knee pain, restricted range of motion, incidents of giving way, swelling, and substantial reduction in strength of the quadriceps. Patellectomy compromises the length of the lever arm of the external apparatus mechanism, thereby causing excessive stress on the knee joint during extension [9, 10]. This ultimately causes early degenerative changes and is therefore a relative contraindication for young individuals [10]. An ideal management protocol for nonunion patellar fracture does not exist, and little literature is available about the results of different approaches. Of many techniques available, we believe that quadricepsplasty with double-tension band wiring is a superior modality of treatment and should be used for patients with such an unusual presentation.

## 4. Conclusion

We conclude that quadricepsplasty with double-tension band wire management is a good surgical measure in treatment of nonunion patella with quadriceps contracture.

## Figures and Tables

**Table 1:** Various studies comparing different modalities of treatment for nonunion patella.

Authors reporting nonunion of patella	Number of cases reported	Number cases treated conservatively	Mean age	Mean duration of delay	Treatment for quadriceps contracture (single-stage or double-stage)	Results (in terms of knee ROM)
Uvaraj et al. [4]	22	—	43 years	3 (range: 2–6.5 months)	No	0 to 110 degrees
Klassen and Trousdale [5]	20	7	38 years	34 months	Yes/single-stage	0 to 109 degrees
Lachiewicz [6]	1	—	67 years	2 years	Yes/single-stage	5 to 80 degrees of flexion
Dhar and Mir [7]	1	—	54 years	1 year	Yes/double-stage	0 to 135 degrees of flexion
Total	44					
